# Correlation of photoreceptor damage with anti-retina antibodies level in aqueous humor in macular edema patients

**DOI:** 10.1038/s41598-022-25875-y

**Published:** 2022-12-08

**Authors:** Xinyao Han, Linqi Zhang, Jiyang Tang, Zongyi Wang, Siying Li, Li Yuan, Jinfeng Qu

**Affiliations:** 1 Department of Ophthalmology, Eye Diseases and Optometry Institute, Peking University People’s Hospital, Beijing, China; 2 Beijing Key Laboratory of Diagnosis and Therapy of Retinal and Choroid Diseases, College of Optometry, Peking University Health Science Center, Beijing, China

**Keywords:** Immunology, Retinal diseases

## Abstract

This study aimed to investigate the correlation between the severity of photoreceptor damage and the level of anti-retina antibodies (ARAs) in aqueous humor, including recoverin, CA II and enolase-α IgG antibody of macular edema patients. Aqueous humor samples were collected from macular edema patients and from cataract patients. Patients were divided into three groups according to the severity of discontinuity of ellipsoid zone (EZ) shown on optical coherence tomography (OCT) imaging: cataract patients with intact EZ, macular edema patients with mild EZ damage, and macular edema patients with severe EZ damage. The level of ARAs was determined with enzyme-linked immunosorbent assay (ELISA). The correlation between the level of ARAs and the degree of photoreceptor damage was analyzed. The level of ARAs of the intact EZ group was significantly lower than that in the severely damaged group (*P* < 0.05). The level of recoverin IgG of the intact EZ group was significantly lower than mildly damaged group (*P* = 0.030). In a subgroup analysis, the level of recoverin IgG of DME patients was correlated with their central retinal thickness (CRT) (*r* = 0.462, *P* = 0.035). The level of ARAs in aqueous humor of patients with DME and RVO-ME was correlated with the degree of photoreceptor damage.

## Introduction

Anti-retina autoantibodies (ARAs) are a class of autoantibodies that bind specifically to retinal proteins. ARAs are often used as a diagnostic tool for autoimmune retinopathies (AIR) which was characterized by rapid progressive outer retinal damage accompanied by visual function impairment^1–3^. However, the specificity of ARAs for AIR had not been determined yet. In recent years, ARAs have been reported to be present in many non-AIR conditions, such as uveitis^4^, retinitis pigmentosa (RP)^5–7^, age-related macular degeneration (AMD)^8–10^, central serous chorioretinopathy (CSC)^11^, diabetic retinopathy (DR)^12–14^ and macular telangiectasia type 2^15^ . It has been reported that the fumarase antibody titers in the blood of diabetic macular edema (DME) patients at baseline are associated with photoreceptor damage and poor response after anti-VEGF treatment^15^. Another ARA, anti-hexokinase 1 IgG in blood, has also been reported to be elevated in diabetic macular edema (DME) patients compared to those patients with DR but without DME^16^.

It is still unclear whether the presence of ARAs is a cause or a consequence of these diseases. There had been postulated that the ARA in the blood can pour into the extravascular space when the blood-retina barrier (BRB) was disrupted and induce neuroinflammation which results in photoreceptor damage. Photoreceptor damage then may trigger more release of retinal antigen and cause more production of ARAs which set up a vicious circle between ARAs and photoreceptor damage. It was still unknown about the correlation between ARAs and many eye diseases or systemic diseases, especially autoimmune diseases.

This study aims to investigate the relationship between the severity of photoreceptor damage and the level of ARAs in aqueous humor, including recoverin, CA II and enolase-α IgG antibody.

## Material and methods

### Patients

This was a cross-sectional study that was conducted following the Declaration of Helsinki and approved by the Ethic Committee of Peking University People’s Hospital. All participants had signed informed consent before the study began. We enrolled a consecutive series of patients on their first time of receiving intravitreal injection for DME or macular edema secondary to retinal vein occlusion (RVO-ME), with interruption or loss of the ellipsoid zone (EZ) band shown on optical coherence tomography (OCT) images in Peking University People’s Hospital from April 2021 to October 2021. Only the eye with poorer visual acuity was included when both eyes met the inclusion criteria. Senile cataract patients undergoing cataract surgery in the same period, with intact EZ shown on OCT images, were recruited as the control group.

Patients were excluded from this study if: (1) patients with a history of intraocular surgery except for phacoemulsification and posterior chamber intraocular lens (IOL) implantation; (2) patients with a history of retinal photocoagulation; (3) patients were diagnosed with uveitis; (4) patients were diagnosed with AMD, AIR, or RP; (5) clear OCT images of the study eye were not applicable; (6) patients were diagnosed with glaucoma or with shallow anterior chambers; (7) patients suffered from systemic disease except diabetes and hypertension (e.g., autoimmune rheumatic disease, hematologic diseases, and tumors); and (8) patients were pregnant or lactating.

### Clinical examination

All enrolled patients underwent complete ophthalmologic examinations, including best-corrected visual acuity (BCVA), intraocular pressure (IOP), slit-lamp examination, dilated fundoscopy, fundus photography (FP), and RTVue-XR Avanti OCT (Optovue, USA) or Cirrus 5000 HD-OCT (Zeiss, Germany). Radial scan or Raster scan protocol passing through the foveal center was used to scan their macular area. Radial scan of RTVue-XR shows 18 radial lines with 8 mm length overlaid on the widefield reference image. Raster scan of Cirrus 5000 shows 25 parallel lines with 6 mm length.

The severity of photoreceptor damage of enrolled eyes was graded according to the length of EZ defect on their OCT images obtained within 7 days. The length of the EZ defect on each B-scan of OCT images was measured using the build-in caliper tool of OCT device and the maximum number was recorded as the EZ defect of this eye. EZ defect length ≥ 1.2 mm was defined as severely damaged (the severely damaged group), and < 1.2 mm was defined as mildly damaged (the mildly damaged group). The measurement and grouping of study eyes were determined independently by two graders (HX, TJ), and discrepancies were adjudicated by another retinal specialist (QJ).

### Sample collection and measurement of ARAs

Aqueous humor samples were drawn from the anterior chamber using an insulin needle before intraocular injections or corneal incisions during cataract surgeries. All samples were stored in sterile centrifuge tubes and frozen at −80 ℃ immediately after collection.

The level of recoverin, CA II and enolase-α IgG antibody of each sample were measured using an enzyme-linked immunosorbent assay (ELISA) kit (Beijing Weilab Biotechnology Co., Ltd., China). All measuring steps were operated in strict accordance with the instruction of the ELISA kits: (1) dilute original density standard and sample; (2) add standard and sample and incubate for 30 min at 37 ℃; (3) wash five times; (4) add HRP-conjugate reagent and incubate for 30 min at 37 ℃; (5) wash five times; (6) add chromogen solution A and B and incubate for 10 min at 37 ℃; (7) add stop solution; (8) read absorbance at 450 nm within 15 min; (9) calculate.

### Statistical analysis

The distribution of continuous variables was assessed using the Shapiro–Wilk test. Continuous variables with normal distribution were presented as means ± standard deviation (SD) and compared by *t*-test or analysis of variance (ANOVA) with Bonferroni correction. Non-normally distributed variables were presented as median (interquartile range) and compared using the Kruskal–Wallis test. Spearman’s correlation coefficient was used to evaluate the statistical correlation. A chi-square test was performed for categorical variables. *P* values < 0.05 were considered statistically significant. SPSS version 23 (SPSS, Chicago, Illinois, USA) was used for statistical analyses.

## Results

50 patients (50 eyes) with macular edema were enrolled in this study, among which 21 were male, and 29 were female, with an average age of 57.40 ± 15.09 years. 19 eyes had mild EZ damage and 31 eyes had severe EZ damage. 30 cataract patients with intact macular were included in the intact EZ group among which 9 were male and 21 were female with an average age of 71.87 ± 9.59 years. The demographic characteristics of all enrolled 80 patients were described in Table 1.

**Table 1:** Baseline characteristics and levels of ARAs in the aqueous humor in different groups.

Parameter	The intact EZ group (*n* = 30)	The mild EZ damage group (*n* = 19)	The severe EZ damage group (*n* = 31)	*F/H/U/χ*^*2*^	*P*
Age (year)	71.87 ± 9.59	57.21 ± 13.44^a^	57.52 ± 16.23^a^	10.938	< 0.001
Sex (male/female)	9/21	6/13	15/16	2.572	0.276
BCVA (logMAR)	0.25 (0.33)	0.20 (0.18)	0.10 (0.15)^a^	7.378	0.025
Length of EZ defect (mm)	0	0.54 ± 0.56^a^	2.52 ± 0.85^a^	148.090	< 0.001
Anti-recoverin (ng/ml)	1.33 (0.26)	1.55 (0.53)^a^	1.80 (0.47)^a^	24.240	< 0.001
Anti-CA II (ng/ml)	5.92 ± 1.09	6.41 ± 1.12	6.81 ± 1.08^a^	5.089	0.008
Anti-enolase-α (ng/ml)	10.07 (2.30)	12.10 (2.14)	13.29 (4.42)^a^	24.297	< 0.001

^a^*P* < 0.05 comparing to the intact EZ group.

The level of recoverin IgG antibody (anti-recoverin) in the aqueous humor was 1.33 (0.26) ng/ml in the intact EZ group, 1.55 (0.53) ng/ml in the mild EZ damage group, and 1.80 (0.47) ng/ml in the severe EZ damage group. The level of anti-recoverin was 1.79 (0.53) ng/ml in DME and RVO-ME patients, higher than the intact EZ group (*P* < 0.001). The level of intact EZ group was much lower than that in the mild EZ damage group (*P* = 0.030) and severe EZ damage group (*P* < 0.001). There was no statistical difference between the mild EZ damage group and the severe EZ damage group (*P* = 0.261).

The level of CA II IgG antibody (anti-CA II) in the aqueous humor was 5.92 ± 1.09 ng/ml in the intact EZ group, 6.41 ± 1.12 ng/ml in the mild EZ damage group, and 6.81 ± 1.08 ng/ml in the severe EZ damage group. The level of anti-CA II was 6.66 ± 1.11 ng/ml in DME and RVO-ME patients, higher than the intact EZ group (*P* = 0.005). The level of anti-CA II was much lower in the intact EZ group than in the severe EZ damage group (*P* = 0.006). There was no statistical difference between the level of anti-CA II in the mild EZ damage group and the intact EZ group (*P* = 0.402), and in the mild EZ damage group and the severe EZ damage group (*P* = 0.615).

The level of enolase-α IgG antibody (anti-enolase) in the aqueous humor of the intact EZ group, the mild EZ damage group, and the severe EZ damage group was 10.07 (2.30) ng/ml, 12.10 (2.14) ng/ml, and 13.29 (4.42) ng/ml respectively. The level of anti-enolase-α was 12.69 (3.17) ng/ml in DME and RVO-ME patients, higher than the intact EZ group (*P* < 0.001). The level of anti-enolase was lower in the intact EZ group than in the severe EZ damage group (*P* < 0.001). There was no statistical difference between the level of anti-enolase in the mild damage group and the control group (*P* = 0.087). There was also no statistical difference between the level of anti-enolase in the mild EZ damage group and the severe EZ damage group (*P* = 0.098).

**Figure 1:**
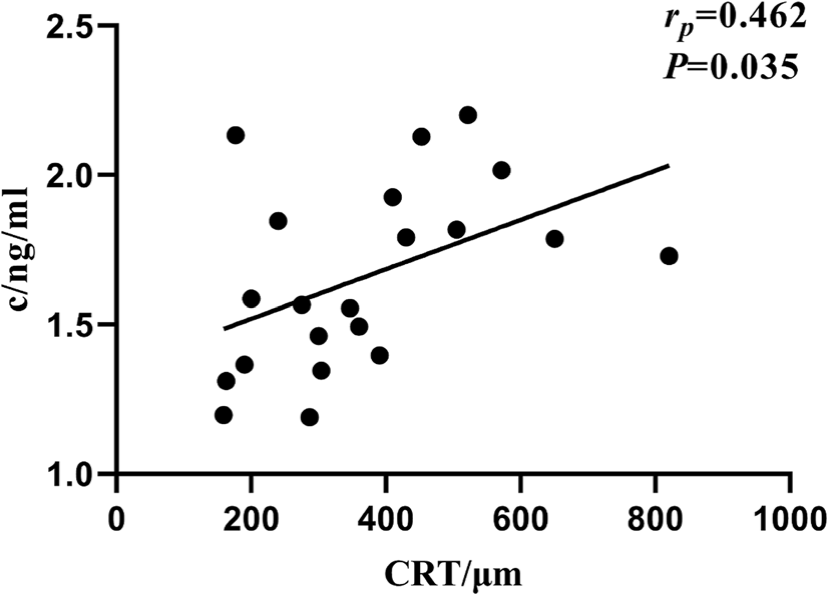
The correlation between the level of anti-recoverin and CRT of DME patients.

There was no statistical difference in the levels of the three ARAs in the aqueous humor between DME and RVO-ME patients (Table 2). Moreover, the level of anti-recoverin in the aqueous humor of DME patients was correlated with the mean macular retinal thickness (CRT) (*r* = 0.462, *P* = 0.035) (Fig. 1).

**Table 2:** Levels of ARAs in the aqueous humor of DME patients and RVO-ME patients.

Disease	Anti-recoverin (ng/ml)	Anti-CA II (ng/ml)	Anti-enolase-α (ng/ml)
DME	1.59 (0.50) (n = 21)	6.41 ± 0.90 (n = 21)	12.48 ± 2.83 (n = 21)
RVO-ME	1.86 (0.55) (n = 29)	6.84 ± 1.22 (n = 29)	12.74 ± 1.77 (n = 29)
*U/t*	251.000	−1.346	0.370
*P*	0.293	0.185	0.714

## Discussion

ARAs can attack any retinal cell type, such as photoreceptor cells, ganglion cells, bipolar cells and was critical for the diagnosis of AIR. However, not all patients who meet the clinical diagnosis of AIR have detectable retinal autoantibodies in their sera. In previous literature reports, this proportion is about 40–65%^17,18^. In addition, the literature reports that retinal autoantibodies can also be found in some normal people and some patients with systemic diseases, such as inflammatory bowel disease, Behcet's disease, systemic lupus erythematosus and multiple sclerosis^19–21^.

Some immunochemical techniques have been used to detect ARAs. The most common technique used for detecting ARAs is western blot (WB). But WB lacks specificity because it confirms the antibody type based on the size of protein. It requires an additional verification using purified native or recombinant proteins to confirm the specificity of ARAs and can be influenced by other factors in the operation process^22,23^. Immunohistochemistry (IHC) can determine the specific binding sites of retinal autoantibodies in the retina and the intensity of staining shows the relative levels of ARAs. But the results of IHC should be confirmed by western blotting or other methods that are more sensitive^22^. The principle of ELISA is antibody-antigen interaction response. The enzyme substrate is applied to produce a colorimetric reaction and the color intensity is positively correlated to the amount of ARAs. It is one of the most sensitive immunoassay methods to detect ARAs but it is not suitable for the detection of unknown antibodies^23^. With differences existing in the protocols of different laboratories and the lack of standardization of sensitivity, specificity, positive predictive values, and negative predictive values, it is difficult to promote further clinical applications of ARAs.

Previous studies on ARAs usually analyzed serum samples, while this study assessed aqueous humor samples. Because of the blood-retinal barrier (BRB), the local production of ARAs in aqueous humor might show a different pattern and clinical importance comparing with serum ARAs. The factors which can damage BRB may associate with the formation of pathological ARAs, such as laser-induced retinal injuries, intraocular surgery, and some diseases^24–26^. Zeng et al. reported that anti-enolase-α antibody and anti-CAII antibody could be observed in the serum of patients with presumed AIR, patients with other retinopathies and healthy donors, while anti-recoverin antibody was only present in patients with cancer-associated retinopathy (CAR). The prevalence of enolase-α antibody and CA II antibody were both approximately 33% in healthy controls^19^. The prevalence of ARAs in aqueous humor has not been reported yet.

Recoverin, a calcium binding protein, is regulating rhodopsin phosphorylation in outer segments (OS) of photoreceptor cells^27^. The antibody of recoverin can enhance rhodopsin phosphorylation levels and increase intracellular calcium levels of retina cells after binding to recoverin^28^. This antibody can activate several apoptotic pathways such as caspase 3 and caspase 9 dependent apoptosis pathway, leading to the death of retinal cells^28,29^. This study found that the level of anti-recoverin in aqueous humor increased after EZ band injury, the level of anti-recoverin of severe EZ damage group was higher than that of mild EZ damage group, but the difference was not statistically significant, which may be related to the small number of subjects in mild injury group.

Enolase-α is an important catalyzing enzyme in the glycolytic pathway. It is widely distributed in the retina and mainly distribute in photoreceptor cells and Müller cells^30^. Enolase-α antibody may penetrate retinal tissue and inhibit the catalytic function of enolase-α, resulting in the dysfunction of glycolysis and ATP depletion in retina cells. Then the level of intracellular calcium is elevated and intracellular pH is decreased^31^. Finally, this antibody eventually leads to the activation of the caspase 3 dependent apoptosis pathway and mitochondrial apoptosis pathway in retinal cells. Enolase-α IgG antibody has been reported in cancer, Alzheimer’s disease (AD), RA and even healthy individuals^31–33^.

CA II is a member of the family of carbonic anhydrases (CAs). CAs family members are responsible for the reversible conversion of carbon dioxide to bicarbonate and participate in ion transportation and pH control^34,35^. CA II antibody can damage cellular function and lead to intracellular pH decreasing through inhibiting the enzymatic activity of CA II. It also increases the level of intracellular calcium and lead to caspase activation in retinal cells. These detrimental effects finally lead to cell death^35^.CA II antibody are also found in some autoimmune diseases such as SLE, Sjögren’s syndrome and systemic sclerosis^19–21^. CA II antibodies can also be detected in healthy people, with a positive rate of about 33%^19^. We did not find difference of Enolase-α and CA II antibody between macular edema patients and control group.

Our study has some limitations. Only limited numbers of patients with DME or RVO-ME were recruited for the study, suggesting possible selection bias. On the other hand, there were only three kinds of ARAs investigated. For more comprehensive analyses, we need large-scale studies integrating more ARAs to confirm the role of ARAs in photoreceptor damage.

In conclusion, we firstly demonstrate that higher titers of ARAs in the aqueous humor are associated with more severe damage of EZ, suggesting that ARAs in aqueous humor may be associated with photoreceptor damage of DME and RVO-ME patients. The use of ARAs as the diagnostic tool for AIR should be treated with caution. Further studies are necessary to confirm these observations in larger cohorts and reveal the role of ARAs in different diseases.

## Data Availability

The datasets generated during and analysed during the current study are available from the corresponding author on reasonable request.
